# Akt3 is responsible for the survival and proliferation of embryonic stem cells

**DOI:** 10.1242/bio.024505

**Published:** 2017-05-08

**Authors:** Ling Wang, Delun Huang, Zongliang Jiang, Yan Luo, Carol Norris, Ming Zhang, Xiuchun Tian, Young Tang

**Affiliations:** 1 Department of Animal Science, Institute for Systems Genomics, University of Connecticut, Storrs, CT 06269, USA; 2 Animal Reproduction Institute, Guangxi University, Nanning, 530004, People's Republic of China; 3 Center for Open Research Resources and Equipment, University of Connecticut, Storrs, CT 06269, USA

**Keywords:** Akt3, Embryonic stem cells, Cell survival, Cell cycle, p53

## Abstract

The phosphatidylinositol 3-kinase (PI3K)/protein kinase B (PKB/Akt) pathway plays an important role in regulating cell proliferation, metabolism, and survival. However, the distinct roles of Akt isoforms (Akt1, Akt2, and Akt3) in pluripotent stem cell maintenance are not fully defined. Using mouse embryonic stem cells (ESCs), we show that direct inhibition of Akt activity leads to ESC apoptosis. The Akt3, but not Akt1 or Akt2, activity specifically regulates this effect. Inhibiting Akt3 also leads to a cell cycle arrest at G1 phase. These regulatory roles of Akt3 are dependent on its kinase activity. Blocking the expression of Akt1 plus Akt2 in ESCs does not affect cell survival or proliferation, although blocking Akt1 aggravates the apoptotic effect induced by depletion of Akt3. We further show that blocking Akt3 in ESCs results in significant nuclear accumulation of p53, as well as the activation of its downstream targets, such as Mdm2, p21, and Fas. Inhibiting p53 and its downstream targets partially rescued the effects caused by Akt3-depletion. Our results revealed an Akt3 isoform-specific mechanism for ESC survival and proliferation involving the control of p53 activity.

## Abstract (precis)

**Summary:** We identified that Akt isoform 3, but not Akt1 or Akt2, specifically regulates embryonic stem cell survival and proliferation. Mechanistically, this is achieved partially through controlling the p53 pathway activity.

## INTRODUCTION

The phosphatidylinositol 3-kinase (PI3K)/protein kinase B (PKB/Akt) pathway plays a central role in mediating extracellular stimuli that controls diverse functions including cell proliferation, growth, survival, and metabolism (Manning and Cantley, 2007; Vanhaesebroeck and Alessi, 2000). Activated by receptor tyrosine kinases, PI3K converts phosphatidylinositol-4,5-bisphosphate (PIP_2_) to phosphatidylinositol-3,4,5-trisphosphate (PIP_3_), which recruits the Akt serine-threonine kinase to the plasma membrane, where Akt is phosphorylated and activated by mammalian target of rapamycin complex 2 (mTORC2) and phosphoinositide-dependent kinase 1 (PDK1) at Ser473 and Thr308, respectively (Alessi et al., 1997; Sarbassov et al., 2005). Activated Akt then phosphorylates its downstream targets to regulate various cellular events. This process can be reverted by phosphatase and tensin homolog (Pten), which dephosphorylates PIP_3_ to PIP_2_, thereby inhibiting Akt activation (Maehama and Dixon, 1998; Paling et al., 2004; Stambolic et al., 1998).

There are three Akt isoforms encoded by different genes, namely Akt1/PKBα, Akt2/PKBβ and Akt3/PKBγ, which all contain conserved peptide sequences between mice and humans (Bellacosa et al., 1991; Brodbeck et al., 1999; Cheng et al., 1992). These three isoforms share a similar N-terminal Pleckstrin-homolog domain, a central serine-threonine kinase domain, and a C-term hydrophobic motif-containing regulatory region characteristic of the protein kinase-A, -G, and -C families (Hanada et al., 2004; Manning and Cantley, 2007; Yang et al., 2004). In mice, both Akt1 and Akt2 mRNAs are expressed ubiquitously in various tissues, with the highest Akt2 expression in insulin-responsive tissues including fat, skeletal muscle and liver (Altomare et al., 1998; Cho et al., 2001a). The expression of Akt3, however, is restricted in selective organs including the brain, testis, lung, mammary gland, and fat (Yang et al., 2003). These expression patterns also correlate with observations from knockout studies, which revealed a general association of Akt1 with organismal growth and cell survival (Chen et al., 2001; Cho et al., 2001b); Akt2 with glucose metabolism (Cho et al., 2001a; Garofalo et al., 2003); and Akt3 with brain development (Tschopp et al., 2005). However, the specific roles of each Akt isoform in different cell types are largely undefined (Chin and Toker, 2011).

Embryonic stem cells (ESCs) hold enormous promise in regenerative medicine because of their pluripotency and capacity for self-renewal. Although it has been reported that in murine ESCs, inhibition of PI3K suppresses and knockout of Pten increases Akt activity and cell proliferation (Jirmanova et al., 2002; Lianguzova et al., 2007; Sun et al., 1999), the functional correlation between direct inhibition of specific Akt isoforms and ESC growth is not understood. At the same time, ESCs share similar properties with cancer stem cells, such as high cell proliferation rates and the presence of many ESC-specific genes and regulatory pathways in various types of tumors (Ben-Porath et al., 2008; Kim and Orkin, 2011; Wong et al., 2008). Isoform-specific Akt abnormalities have been reported in many cancer cells, such as the overexpression of Akt1 in thyroid and non-small cell lung cancers (Lee et al., 2011; Saji et al., 2011), Akt2 and Akt3 in malignant glioma (Mure et al., 2010; Turner et al., 2015; Zhang et al., 2009), and Akt3 in melanoma, ovarian, and breast cancer (Chin et al., 2014; Grabinski et al., 2014; Madhunapantula et al., 2011; Stahl et al., 2004; Turner et al., 2015). The underlying mechanism of these Akt isoform dysregulations, however, is largely unclear. Therefore, understanding Akt isoform-specific regulation in ESCs may provide important insight not only for stem cell therapy, but also for cancer target identification and treatment.

In this study, we investigated the specific roles of the Akt isoforms in mouse ESCs. We report here an Akt3-dependent ESC survival and proliferation mechanism, which involves suppressing the p53 activity.

## RESULTS

### Blocking Akt activity results in ESC apoptosis

**Fig. 1.**
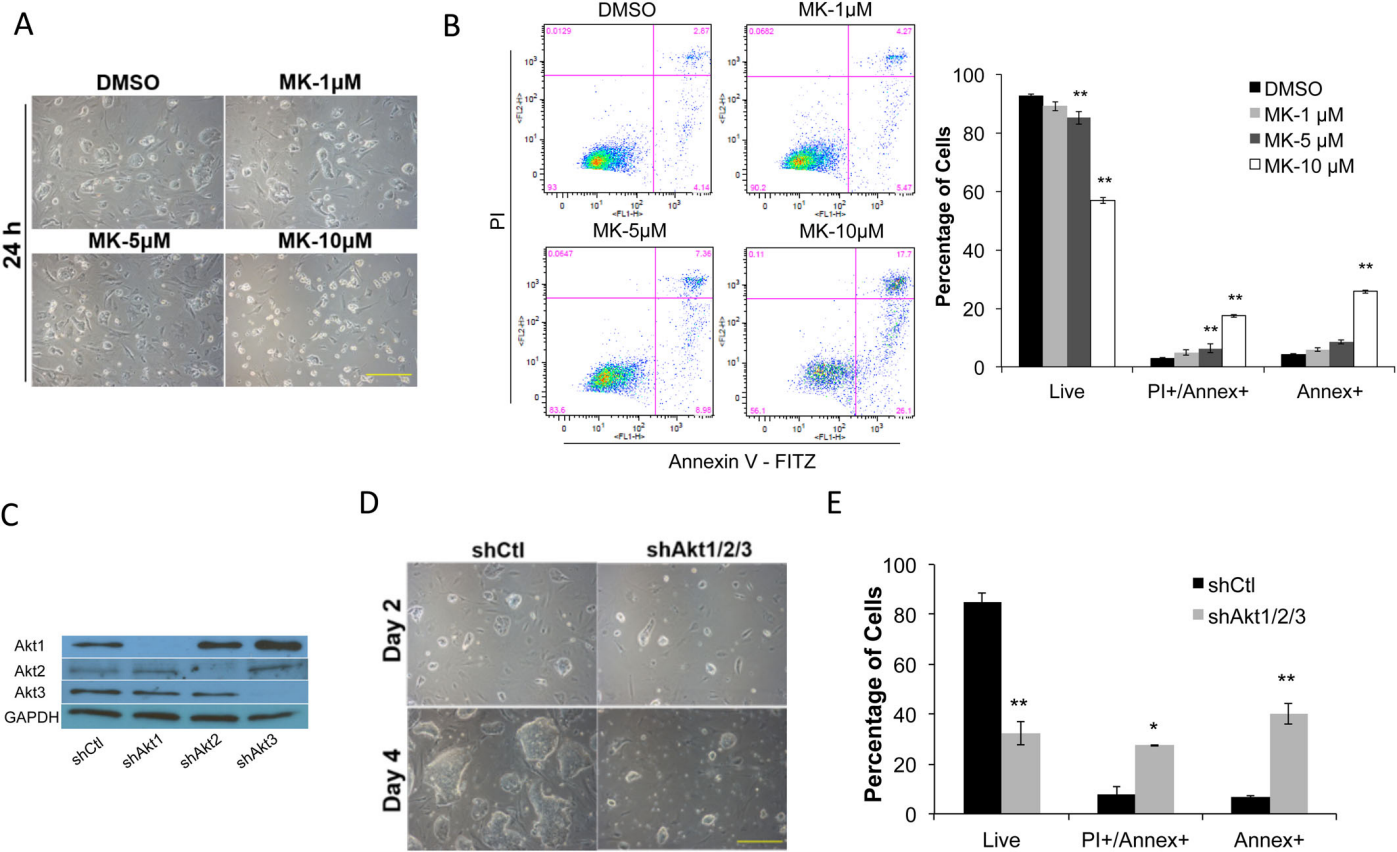
**Blocking Akt activity leads to ESC apoptosis.** (A) R1-ESCs grown in 2i/LIF medium plus DMSO control or 1–10 µM of MK for 24 h. Scale bar: 250 µm. (B) R1 ESCs treated as described in A were incubated with Annexin V-FITC (Annex) and Propidium Iodide (PI) for 30 min and analyzed by flow cytometry. Percentages of late apoptotic/necrotic (Annex+/PI+), early apoptotic (Annex+), and live cells (unstained) are shown (data shown are mean±s.d., **P<0.01, *n*=3). (C) Western blot of total protein extracts from ESCs 3 days after lentiviral control (shCtl), shAkt1, shAkt2, and shAkt3 transduction, respectively. Proteins were blotted with the respective Akt antibodies with GAPDH used as a loading control. (D) ESCs expressing lentiviral shCtl or a combination of shAkt1, shAkt2, and shAkt3 (shAkt1/2/3) for 2 and 4 days, respectively, in 2i/LIF medium. Scale bar: 250 µm. (E) ESCs treated as described in D were incubated with Annex and PI for 30 min and analyzed by flow cytometry. Percentage of early, late apoptotic and live cells were determined as described in B (data shown are mean±s.d., *P<0.05, **P<0.01, *n*=2).

We used MK2206 (MK), an Akt-specific allosteric inhibitor (Hirai et al., 2010), to determine the effect of a direct Akt inhibition on the growth of ESCs. Mouse R1 ESCs were grown in 2i/leukemia inhibitory factor (LIF) medium (Silva et al., 2008; Ying et al., 2008), with dual inhibition to the extracellular-signal-regulated kinases 1/2 and the glycogen synthase kinase 3-beta, to study Akt signaling with minimum extracellular stimuli. Within 24 h of applying MK (0–10 µM) to 2i/LIF medium, we observed a dose-dependent inhibition to ESC colony growth, and obviously detached, dead cells in the medium (Fig. 1A). Annexin V apoptosis assay by flow cytometer revealed a significant increase in cell apoptosis and decrease in live cell population at 5 and 10 µM MK levels (Fig. 1B).

We then asked if inhibiting Akt expression by short hairpin RNA (shRNA) could produce the same effect. We previously reported Akt isoform-specific knockdown in mouse embryonic fibroblasts (MEFs) using lentiviral shRNA constructs: shAkt1, shAkt2, and shAkt3 (for maximum Akt3 knockdown, shAkt3 consists of two constructs: shAkt3a and shAkt3b targeting different coding regions of Akt3) (Tang et al., 2014). We first tested the effectiveness of these constructs in ESCs. Western blot analysis revealed barely detectable levels of Akt isoform 1, 2, or 3, three days after the lentiviral shAkt1, shAkt2, or shAkt3 infection, respectively (Fig. 1C). To block all Akt isoform expression, we seeded R1-ESCs at a clonogenic density at day 0, together with the lentiviral shAkt1 plus shAkt2 and shAkt3 (shAkt1/2/3). Similar to the MK treatment, Akt knockdown led to obvious cell detachment, with much smaller colonies compared to the control (shCtl) at day 4 (Fig. 1D). Annexin V analysis again confirmed significantly increased cell apoptosis and decreased live cell population (Fig. 1E). Thus, results from MK and shRNAs confirmed an essential role of Akt activity in ESC survival.

### Akt3, but not Akt1 or Akt2, is responsible for ESC survival

**Fig. 2.**
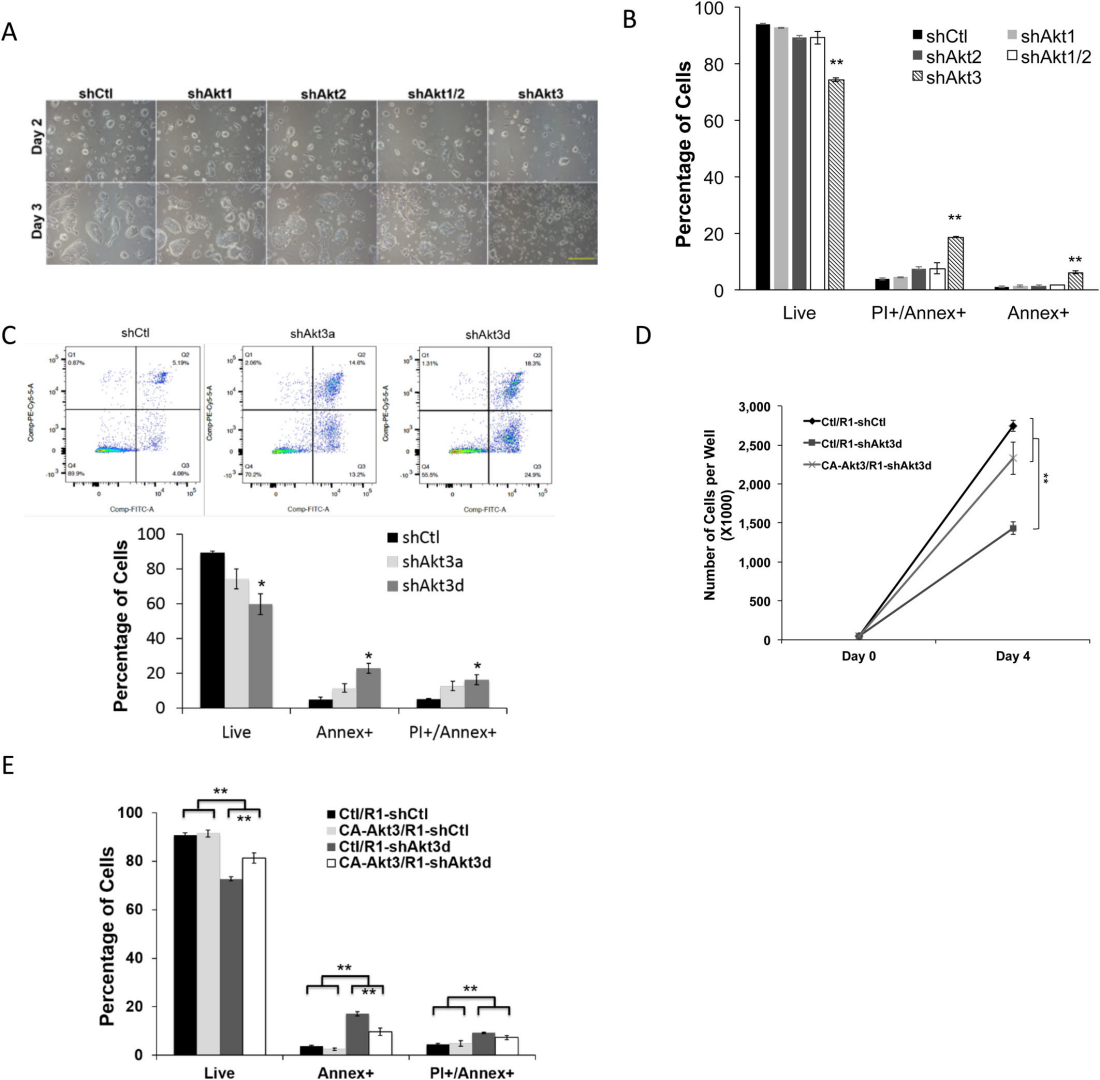
**Blocking Akt3 activity specifically leads to ESC apoptosis.** (A) R1-ESCs expressing lentiviral shCtl, shAkt1, shAkt2, shAkt1 and 2 (shAkt1/2), or shAkt3 for 2 and 3 days and cultured in 2i/LIF medium. Scale bar: 250 µm. (B) ESCs treated as described in A for 3 days were incubated with Annex and PI for 30 min and analyzed by flow cytometry. Percentages of early and later apoptotic cells and live cells are shown (data shown are mean±s.d., **P<0.01, *n*=3). (C) R1-ESCs expressing lentiviral shCtl, shAkt3a, or shAkt3d construct for 3 days were incubated with Annex and PI for 30 min and analyzed by flow cytometry (data shown are mean±s.d., *P<0.05, *n*=2). (D) R1-ESCs overexpression CA-Akt3 (CA-Akt3/R1) or a vector control (Ctl/R1) were treated with lentiviral shCtl or shAkt3d on day 0 and grown for 4 days with cell numbers counted (data shown are mean±s.d., **P<0.01, *n*=2). (E) ESCs treated as in D and grown for 3 days were subjected to Annexin V apoptosis assay. Percentages of early and later apoptotic as well as live cells are shown (data shown are mean±s.d., **P<0.01, *n*=2).

We then examined which Akt isoform functions specifically to improve the survival of ESCs. Using lentiviral shRNAs, we either individually blocked each isoform or Akt isoform 1 plus 2 (shAkt1/2). At day 3 of viral infection, we found that blocking Akt3, but not Akt1, Akt2, or Akt1 plus 2, resulted in an inhibition of colony growth and obvious floating, dead cells (Fig. 2A). Annexin V analysis further confirmed that a specific knockdown of Akt3, but not Akt1 and 2, led to significant apoptosis (Fig. 2B). As shAkt3 constructs (containing shAkt3a and -3b) target only the coding region of Akt3, we developed another lentiviral construct – shAkt3d to specifically target its 3′-untranslated region, and observed a similar apoptotic effect upon applying it to ESCs (Fig. 2C). To further confirm the specificity of our shRNA experiment, we pre-expressed a myristoylated, constitutively active Akt3 (CA-Akt3) in ESCs (CA-Akt3/R1), and inhibited endogenous Akt using lentiviral shAkt3d. Compared to the vector control, CA-Akt3 led to an obvious recovery to the number of attached ESCs (Fig. 2D). This was correlated with partially but significantly rescued cell apoptosis caused by Akt3-knockdown (Fig. 2E). Thus, among the three Akt isoforms, the expression of Akt3 is specifically responsible for regulating the survival of ESCs.

### Akt3 specifically regulates G1/S cell cycle transition in ESCs

**Fig. 3.**
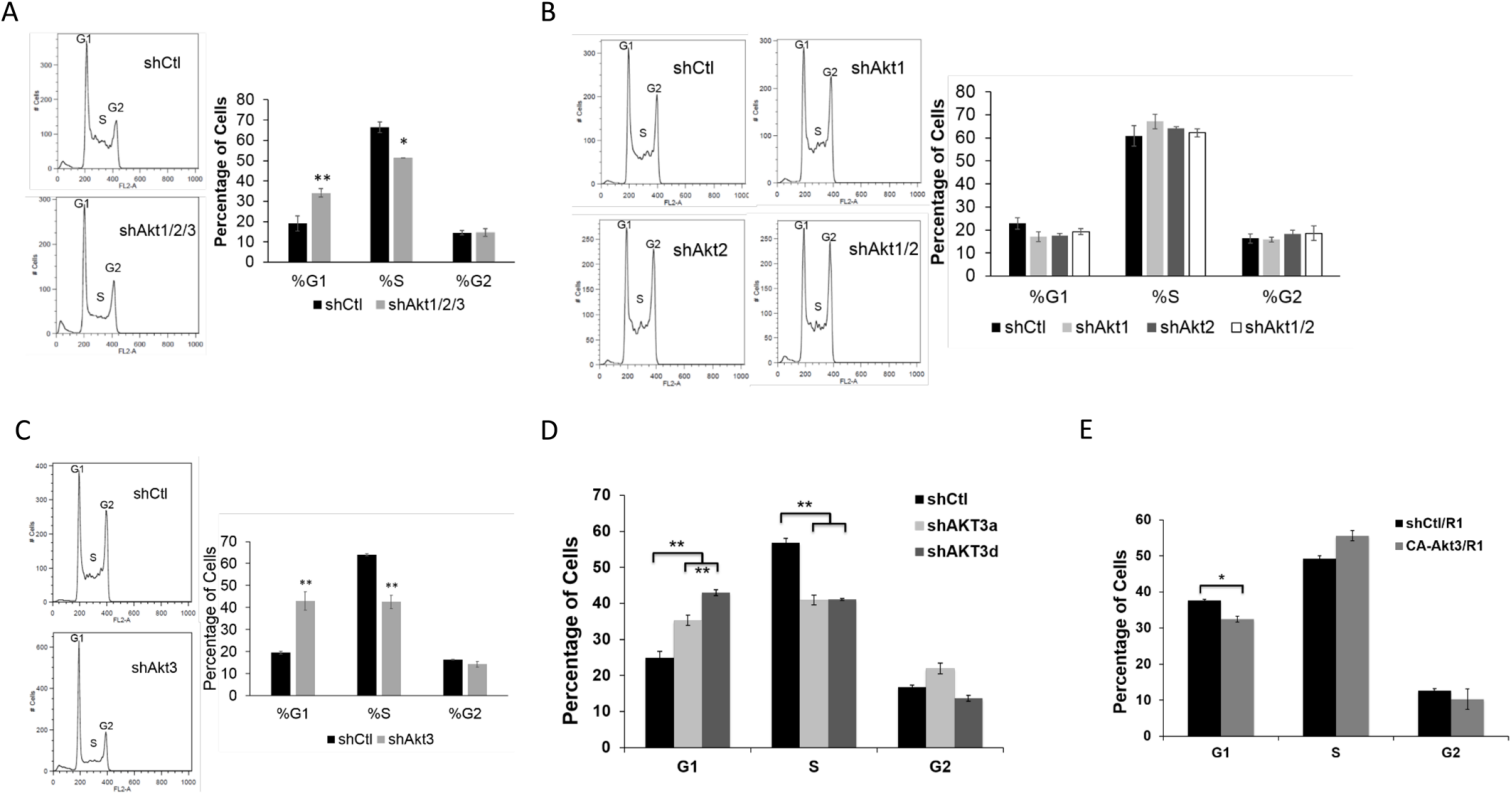
**Akt3 is responsible for G1/S transition in ESCs.** (A) ESCs expressing lentiviral shCtl or shAkt1/2/3 for 3 days were incubated with PI and RNase for 30 min and subjected to cell cycle analysis by flow cytometry. The percentages of cell in G1, S, and G2 phases are shown on the right (data shown are mean±s.d., *P<0.05, **P<0.01, *n*=2). (B) Cell cycle analysis of R1-ESCs treated with shCtl, shAkt1, shAkt2, or shAkt1 plus 2 (shAkt1/2) for 3 days and cultured in 2i/LIF medium. The percentages of cell in G1, S, and G2 phases are shown on the right (data shown are mean±s.d., *n*=2). (C) Cell cycle analysis of R1-ESCs treated with shCtl or shAkt3 for 3 days and cultured in 2i/LIF medium. The percentages of cells in G1, S, and G2 phases are shown on the right (data shown are mean±s.d., **P<0.01, *n*=2). (D) Cell cycle analysis of R1-ESCs treated with shCtl, shAkt3a, or shAkt3d for 3 days and cultured in 2i/LIF medium. The percentages of cells in G1, S, and G2 phases are shown on the right (data shown are mean±s.d., **P<0.01, *n*=2). (E) Cell cycle analysis of R1-ESCs overexpressing CA-Akt3 (CA-Akt3/R1) or a vector control (Ctl/R1). The percentages of cells in G1, S, and G2 phases are shown (data shown are mean±s.d., *P<0.05, *n*=2).

Akt1 and Akt2 activities have been reported to play roles for G1/S and G2/M transition in cancer and other somatic cells (Hers et al., 2011; Toker and Marmiroli, 2014). To confirm a direct Akt activity on ESC cell cycle progression, we first blocked all three Akt isoforms with the lentiviral shAkt1/2/3, and specifically monitored the attached cells at day 3 using propidium iodine (PI) and flow cytometry (Pozarowski and Darzynkiewicz, 2004). A significant increase was observed for cell population at G1 (34% versus ∼20%) by Akt-knockdown (Fig. 3A). This confirms a requirement of Akt activity for the G1/S transition in ESCs. Subsequently, we asked which Akt-isoform deletion might be responsible for the observed G1-arrest. We individually blocked these Akt isoforms using lentiviral shRNAs and found that blocking Akt1, Akt2, or Akt1 plus 2 had no significant impact on the cell cycle progression of ESCs (Fig. 3B). However, blocking Akt3 alone by various lentiviral constructs resulted in significant G1-arrest (Fig. 3C,D). We then wondered whether enhanced activation of Akt3 would promote the cell cycle progression. Indeed, CA-Akt3 overexpressing ESCs exhibited decreased G1 cell population, although there was no significant change to cell viability, compared to the control cells (Fig. 3E and Fig. 2E). Thus, these data demonstrated that the Akt3 isoform specifically promotes G1/S transition in ESCs, in addition to its role in cell survival.

We also cultured ESCs in 20% FBS supplemented with LIF, and examined the effect of individual Akt isoform knockdown to the growth of colonies. Again we observed obvious cell death and reduced colony sizes that are specific to Akt3 depletion similarly as in 2i/LIF medium (Fig. S1). Thus it is clear that the Akt3-dependent ESC survival and proliferation is not restricted to the 2i/LIF medium used in this study.

### Akt3 regulates ESC survival and proliferation through its kinase activity

**Fig. 4.**
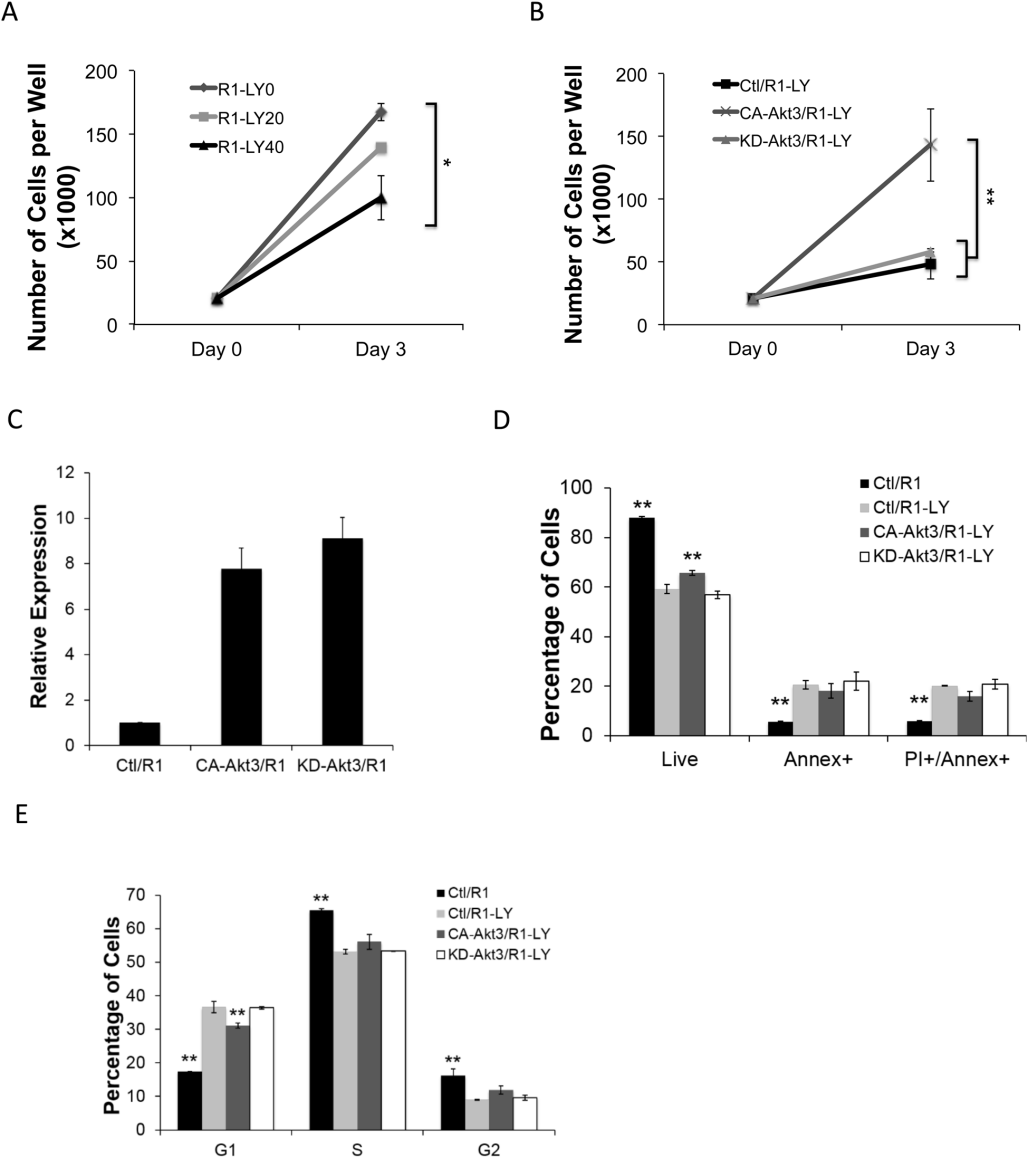
**Akt3 mediates ESC survival and proliferation through its kinase activity.** (A) R1-ESCs were seeded in 12-well plates and grown in 2i/LIF medium on day 0, treated with 0 μM, 20 μM or 40 μM LY294002 (LY0, LY20, or LY40) on day 2 and cell number counted 24 h later (day 3) (data shown are mean±s.d., *P<0.05, *n*=2). (B) R1-ESCs expressing pMCs vector control (Ctl/R1), CA-Akt3 (CA-Akt3/R1), or KD-Akt3 (KD-Akt3/R1) were seeded in 12-well-plates and grown in 2i/LIF medium on day 0, cultured with 40 μM LY294002 (LY) on day 2 and cell number counted 24 h later (day 3) (data shown are mean±s.d., **P<0.01, *n*=2). (C) qRT-PCR analysis of RNAs from Ctl/R1, CA-Akt3/R1, and KD-Akt3/R1 ESCs with primers amplifying both ectopic and endogenous Akt3 expression. All values were normalized to GAPDH and relative to the expression of Ctl/R1 ESCs (data shown are mean±s.d.). (D) R1-ESCs expressing pMCs vector control (Ctl/R1), and ESCs treated as described in (B) with LY for 3 days were incubated with Annex and PI on day 3 for 30 min and analyzed by flow cytometry. Percentages of live, early and later apoptotic cells are shown (data shown are mean±s.d., **P<0.01, *n*=2). (E) R1-ESCs expressing pMCs vector control (Ctl/R1), and ESCs treated as described in B with LY for 3 days were incubated with PI and RNase for 30 min and subjected to cell cycle analysis. The percentages of cell in G1, S, and G2 phases are shown (data shown are mean±s.d., **P<0.01, *n*=2).

Inhibiting PI3K, the upstream Akt kinase activator, results in cell cycle arrest and apoptosis in ESCs (Jirmanova et al., 2002; Lianguzova et al., 2007). However, a kinase-independent function of Akt was also reported for cell survival in cancer cells (Vivanco et al., 2014). We wondered whether the Akt3-mediated ESC survival and proliferation is dependent on Akt kinase activity. To this end, we mutated the ATP binding site of Akt3 with the lysine 177 changed to methionine (KD-Akt3) at a position comparable to the reported Akt1 kinase-dead mutant K179M (Bellacosa et al., 1993; Franke et al., 1995). ESCs stably expressing KD-Akt3 did not show reduced cell growth compared to the control (Fig. S2A). We suspected that the endogenous Akt kinase activity in ESCs might compensate for the KD-Akt3. We then used the PI3K inhibitor LY294002 (LY) to minimize the activation of the endogenous Akt kinase in ESCs. 40 μM LY treatment significantly inhibited the growth of R1 ESCs (Fig. 4A), induced cell apoptosis and G1-arrest similar to that reported previously (Jirmanova et al., 2002; Lianguzova et al., 2007) and similar to that of Akt3-knockdown here (Fig. S2B,C; Fig. 2C and Fig. 3D). We then examined the growth of ESCs under the LY treatment and overexpressing CA-Akt3 or KD-Akt3. A partial CA-Akt activity would be expected here, as reduced PIP_3_ production caused by LY inhibition of PI3K will impair the ability of PDK1 to phosphorylate Akt (Primo et al., 2007). Despite the LY treatment, ESCs overexpressing CA-Akt3 showed significant improvement on cell growth, while the KD-Akt3 expressing ESCs did not improve on total cell number compared to the vector control (Fig. 4B), even though both expressed similar levels of retroviral Akt3 (Fig. 4C). Annexin V and cell cycle assay further revealed that CA-Akt3 expression partially but significantly rescued the apoptosis and G1-arrest caused by LY treatment, whereas the expression of KD-Akt did not show any rescue effect (Fig. 4D,E). Therefore, we conclude that the regulatory role of Akt3 on ESC survival and proliferation is largely, if not all, dependent on its kinase activity.

### Akt3 regulates p53 protein expression in ESCs

**Fig. 5.**
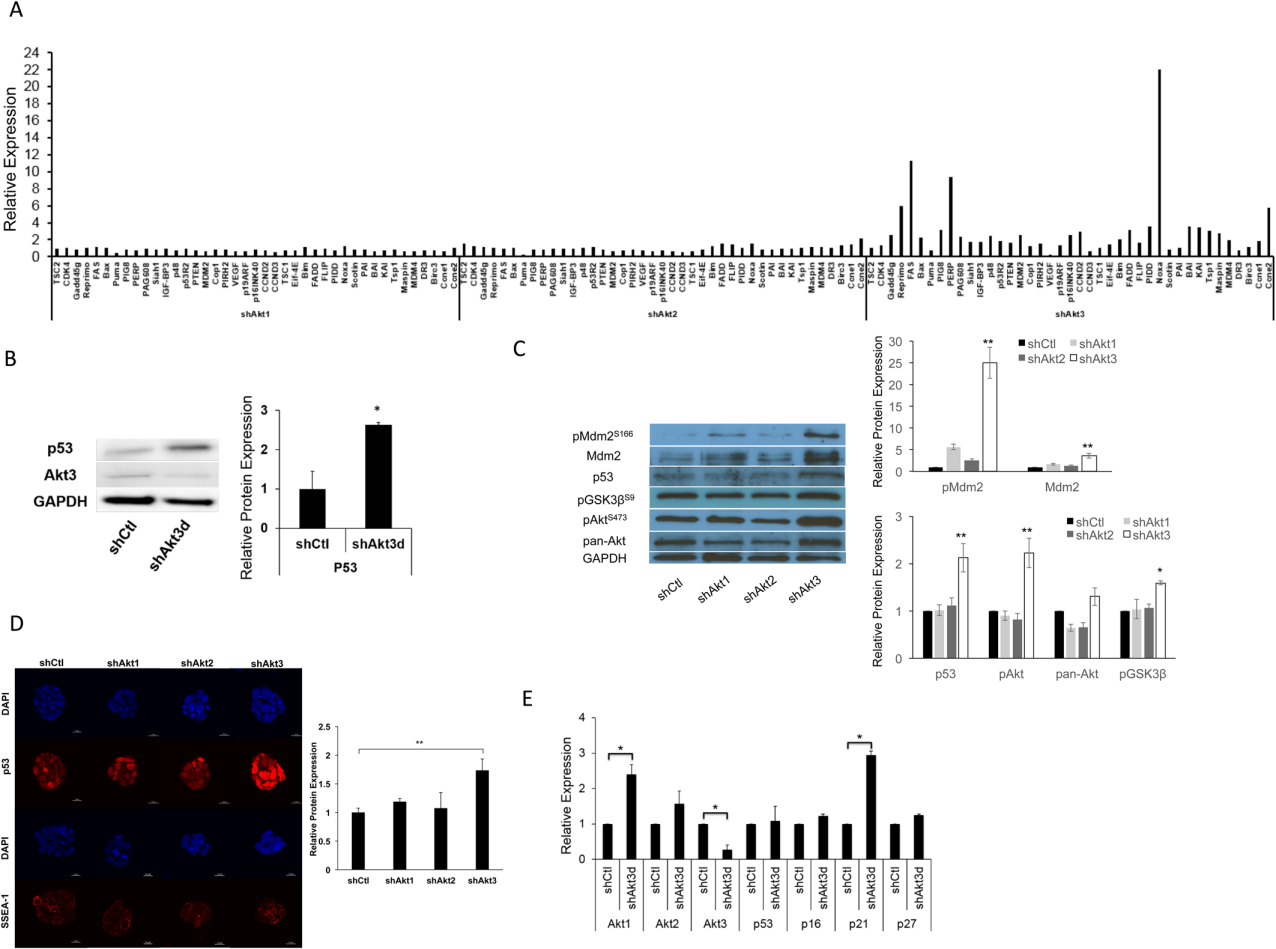
**Akt3 knockdown results in increased p53 activity post-transcriptionally.** (A) qRT-PCR analysis of RNAs from R1-ESCs treated with lentiviral shCtl, shAkt1, shAkt2, or shAkt3 for 3 days and cultured in 2i/LIF medium. All values were normalized to GAPDH and relative to the expression of shCtl ESCs. (B) Western blot of whole protein extracts from ESCs treated with lentiviral shCtl or shAkt3d and grown for 3 days. The specific proteins probed were p53 and Akt3. Protein levels of p53 were quantified using the BioRad Gel Doc Densitometry software. All values were normalized to GAPDH and relative to the expression of shCtl ESCs (data shown are mean±s.d., *P<0.05, *n*=2). (C) Western blot of whole protein extracts from ESCs treated with lentiviral shCtl, shAkt1, shAkt2, or shAkt3 for 3 days and cultured in 2i/LIF medium. The specific proteins probed were phospho (*P*) Mdm2, Mdm2, p53, pGSK3β, pAkt, and pan-Akt. Protein levels of pMdm2, Mdm2, p53, pAkt, pan-Akt, and pGSK3β were quantified using the ImageJ software. All values were normalized to GAPDH and relative to the expression of shCtl ESCs (data shown are mean±s.d., *P<0.05, **P<0.01, *n*=2). (D) Immunostaining of ESCs treated as described in (C). Cells were stained with antibodies for p53 and the stem cell surface marker SSEA-1. Cell nuclei were counterstained with DAPI. Scale bar: 10 µm. Fluorescence signals under each condition were quantified using the ImageJ software, the mean ratios of p53 and DAPI relative to the control ESCs were shown (data shown are mean±s.d., **P<0.01, *n*=5). (E) qRT-PCR analysis on RNAs from R1-ESCs treated with either shCtl, shAkt1, shAkt2, or shAkt3d lentiviruses for 3 days and cultured in 2i/LIF medium. All values were normalized to GAPDH and relative to ESCs treated with shCtl (data shown are mean±s.d., *P<0.05, *n*=3).

We wondered which downstream pathway might be responsible for Akt3-dependent ESC survival and G1/S transition. We examined 36 genes whose expressions are regulated by various Akt downstream targets (Fig. 5A). Knockdown of Akt3, but not Akt1 or 2, showed a marked increase in the expression of p53 responsive genes related to apoptosis and cell cycle control, such as Reprimo (Ohki et al., 2000), Fas (Müller et al., 1998), p53 apoptosis effector related to PMP-22 (Perp) (Attardi et al., 2000), Noxa (Oda et al., 2000), and the p53 direct target and negative regulator mouse double minute 2 homolog (Mdm2) (Barak et al., 1993; Juven et al., 1993; Perry et al., 1993) (Fig. 5A). These data indicate that depletion of Akt3 may lead to the activation of p53 in ESCs. We therefore asked whether the expression of p53 would vary upon Akt3 knockdown. Western blot analysis revealed that blocking Akt3 by different shRNAs, but not blocking Akt1 or -2, led to increased p53 protein expression (Fig. 5B,C). This was also accompanied by an increase in the protein level of Mdm2, the direct p53 target (Fig. 5C). Also, the level of p53 protein was not affected with a dual-depletion of Akt1 and -2 (Fig. S3, left panel), whereas an additional knockdown of Akt3 stimulated p53 (Fig. S3, right panel). Furthermore, immunostaining of p53 protein revealed a significant accumulation of p53 in ESC nucleus, which depends on the depletion of Akt3, but not Akt1 or Akt2 (Fig. 5D).

Although we found that Akt3 specifically regulates p53 at the protein level, we did not detect an increase in p53 mRNA upon Akt3-depletion in ESCs (Fig. 5E). In addition to the increased expression of the p53 targets mentioned above, we also found the up-regulation of p21 (Fig. 5E), a major p53 direct target involved in p53-dependent G1 arrest (Brugarolas et al., 1995; Deng et al., 1995). Taken together, our data indicate that in ESCs, Akt3 specifically suppresses p53 protein activity post-transcriptionally.

### Akt1 deletion aggravates the apoptosis induced by Akt3 knockdown in ESCs

Interestingly, although blocking Akt1 plus -2 in ESCs by shAkt1/2 did not affect ESC survival and proliferation (Fig. 2B and Fig. 3B), we had noticed that ESCs with Akt3 depletion increased the expression of the other two Akt isoforms, especially Akt1 at both protein (Fig. 1C) and mRNA levels (Fig. 5E). This is correlated with an increase of phospho-pan-Akt (pAkt^S473^), and the increased levels of phospho-GSK3β (pGSK3β^S9^) and phospho-Mdm2 (pMdm2^S166^), a direct indication of increased Akt kinase activity (Alarcon-Vargas and Ronai, 2002; Cross et al., 1995; Ogawara et al., 2002) (Fig. 5C).

**Fig. 6.**
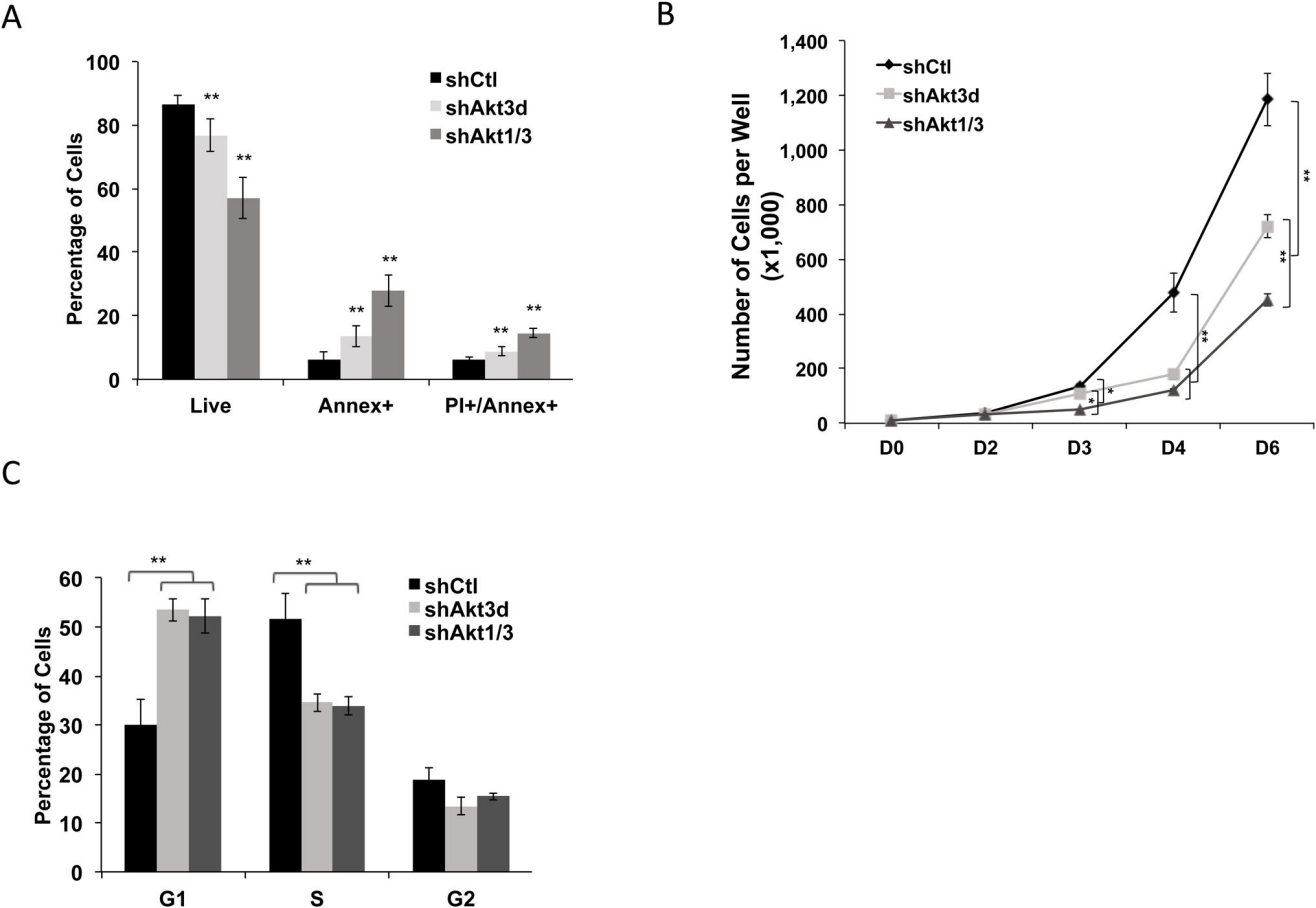
**Depletion of Akt1 aggravates ESC apoptosis induced by Akt3 knockdown.** (A) R1-ESCs expressing lentiviral shCtl, shAkt3d, shAkt1 and -3d (shAkt1/3) for 3 days were incubated with Annex and PI for 30 min and analyzed by flow cytometry. Percentages of early and later apoptotic cells and live cells are shown (data shown are mean±s.d., **P<0.01, *n*=3). (B) R1-ESCs were transduced with lentiviral shCtl, shAkt3d, or shAkt1/3 at day 0, and cell numbers were counted at day 2, 3, 4, 6 with Trypan blue solution under the microscope (data shown are mean±s.d., *P<0.05, **P<0.01, *n*=2). (C) R1-ESCs expressing lentiviral shCtl, shAkt3d, or shAkt1/3 for 3 days were incubated with PI and RNase for 30 min and subjected to cell cycle analysis. The percentages of cell in G1, S, and G2 phases are shown (data shown are mean±s.d., **P<0.01, *n*=2).

As we had observed that not all ESC population underwent apoptosis upon Akt3-knockdown (as indicated by the percentage of live cells) through our experiments, the increased Akt1 expression in Akt3-depleted ESCs may thus reflect a cell response to maintain survivability. We therefore examined whether a concurrent Akt1 inhibition will produce more prominent ESC apoptosis than inhibiting Akt3 alone. Indeed a combined Akt1/3 knockdown resulted in greater apoptosis rate in ESCs compared to targeting Akt3-only (Fig. 6A), even though depleting Akt1 alone had no apoptotic effect (Fig. 2B). Consequently, we observed a greater reduction to the number of attached, viable ESCs by combined Akt1/3 knockdown than by depleting Akt3 only at 3 and 6 days after lentiviral shRNA infection (Fig. 6B). These data are in agreement with the previous *in vivo* studies showing that Akt1^−/−^/Akt3^−/−^ mice were embryonic lethal whereas Akt3^−/−^ mice were viable postnatally (Tschopp et al., 2005; Yang et al., 2005). However, a combined Akt1/3 knockdown induced no additional increase in G1 arrest than the depletion of Akt3 alone in ESCs (Fig. 6C). Taken together, our data demonstrated that blocking Akt1 expression can exacerbate the apoptosis induced by Akt3 knockdown in ESCs, and indicated a compensatory role for ESC survival by increased Akt1 activity in the event of Akt3-depletion.

### p53 activation is a critical event for Akt3 regulated ESC survival and proliferation

**Fig. 7.**
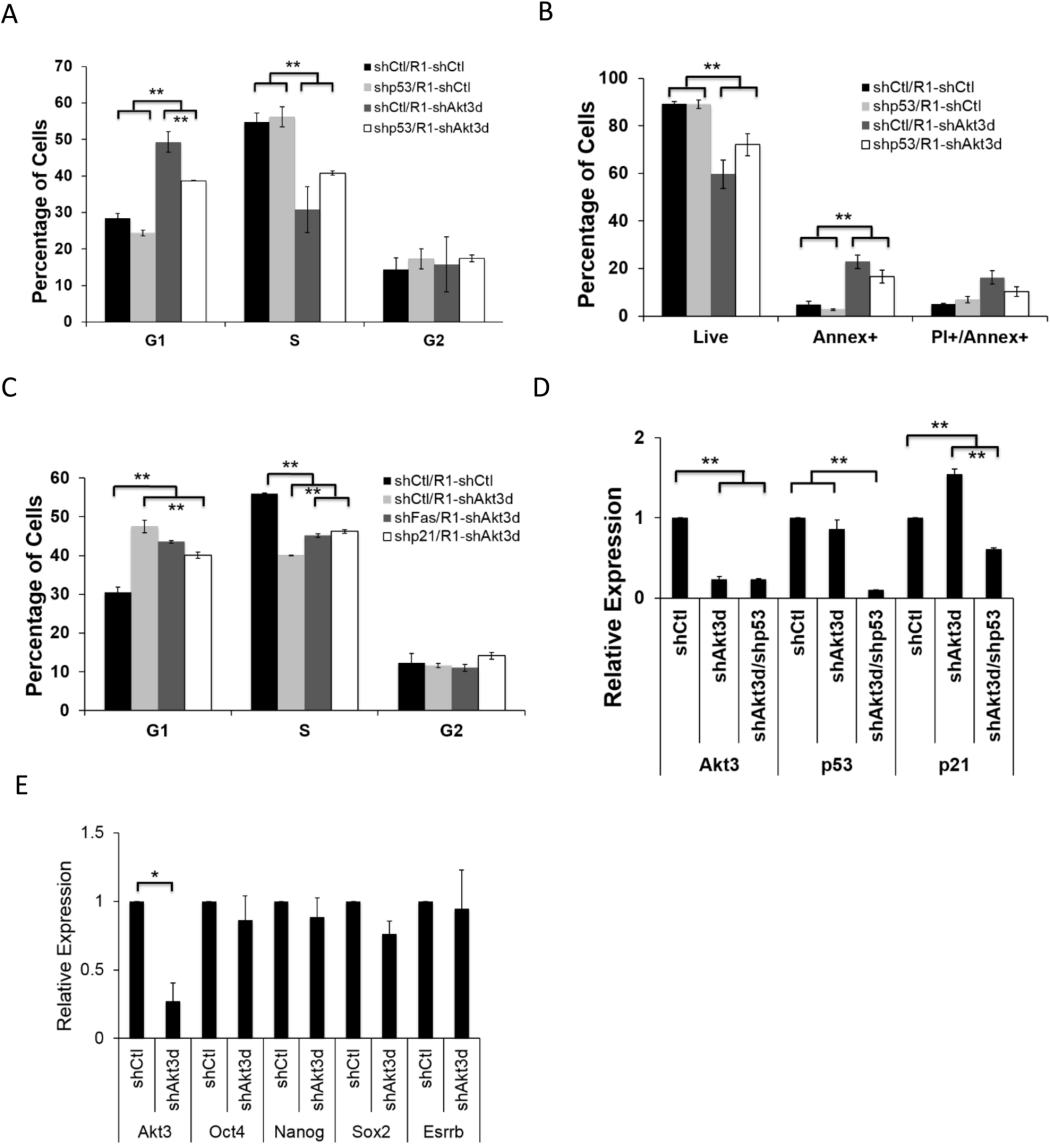
**p53 activation is critical for Akt3-depletion inflicted G1-arrest and apoptosis.** (A) R1-ESCs expressing pLKO.1 vector control (shCtl/R1) or shp53 (shp53/R1) were infected with lentiviral shCtl or shAkt3d on day 0, cells were grown for 3 days, then incubated with PI and RNase for 30 min and subjected to cell cycle analysis. The percentages of cells in G1, S, and G2 phases are shown (data shown are mean±s.d., **P<0.01, *n*=2). (B) ESCs treated as described in A were incubated with Annex and PI for 30 min and analyzed by flow cytometry. Percentages of live, early, and late apoptotic cells are shown (data shown are mean±s.d., **P<0.01, *n*=2). (C) R1-ESCs expressing pLKO.1 vector control (shCtl/R1), shFas (shFas/R1), or shp21 (shp21/R1) were infected with lentiviral shCtl or shAkt3d on day 0, grown for 3 days, then incubated with PI and RNase for 30 min and subjected to cell cycle analysis. The percentages of cells in G1, S, and G2 phases are shown (data shown are mean±s.d., **P<0.01, *n*=2). (D) qRT-PCR Analysis of RNAs from R1-ESCs treated by shCtl, shAkt3d, or shAkt3d plus shp53 for 72 h. All values were normalized to GAPDH and relative to ESCs treated with shCtl (data shown are mean±s.d., **P<0.01, *n*=2). (E) qRT-PCR Analysis of RNAs from R1-ESCs treated by either shCtl, shAkt1, shAkt2, or shAkt3d for 72 h. All values were normalized to GAPDH and relative to ESCs treated with shCtl (data shown are mean±s.d., *P<0.05, *n*=3).

As we discovered that knockdown of Akt3 leads to increased p53 protein expression, we asked whether inhibiting p53 expression might reverse the effects seen in Akt3 depletion. We used a lentiviral construct against p53 (shp53) with more than 90% knockdown efficiency in both MEFs and ESCs (Fig. S4A). R1-ESCs were transduced with lentiviral shp53 (shp53/R1) or shCtl (shCtl/R1) and pre-selected with puromycin for 3 days. These cells (shp53/R1 and shCtl/R1) were then seeded at clonogenic density and transduced with lentiviral shCtl or shAkt3d. We found that compared to the shCtl/R1 cells, inhibiting p53 in ESCs (shp53/R1) partially rescued the G1-arrest and apoptosis caused by shAkt3d infection (Fig. 7A,B), although we also observed a near complete rescue of apoptosis when only a mild degree of cell apoptosis was induced (Fig. S4B,C).

As p21 functions to mediate p53-dependent G1 arrest (Brugarolas et al., 1995; Deng et al., 1995), we asked whether the increased p21 expression upon Akt3 depeletion (Fig. 5E) is responsible for the ESC G1 arrest observed here. We generated ESCs with ∼70% p21 knockdown (shp21/R1, Fig. S4D), and upon Akt3-depletion using shAkt3d, we found that shp21/R1 cells exhibited partial but significant rescue of G1 arrest induced, similar to that of p53-knockdown (Fig. 7A,C). We also measured the p21 expression level with a double inhibition of Akt3 and p53. Akt3-knockdown alone increased while a concomitant knockdown of p53 decreased the p21 expression (Fig. 7D), thus confirming the p53-dependent increase of p21 expression upon Akt3 depletion in ESCs. Knockdown of another p53 target, Fas, in ESCs (shFas/R1) also showed partial rescue of the G1 arrest, although at a lesser degree than the p21 knockdown (Fig. 7C). These data further confirmed that the control of p53 pathway activity by Akt3 is a critical event for ESC survival and proliferation, alongside the other mechanism(s) to be elucidated.

Artificially activated Akt1 can sustain pluripotency of mouse ESCs without LIF (Watanabe et al., 2006). Also, in human ESCs, Akt prevents differentiation by mitigating Smad2/3-mediated Activin-A signaling (Singh et al., 2012). We wondered if the knockdown of Akt3 may cause ESC differentiation, therefore leading to restricted cell proliferation observed in 2i/LIF medium here. qRT-PCR analysis revealed that there was no significant change to the expression of key pluripotency markers, including Oct4, Sox2, Nanog, and Esrrb, upon Akt3 knockdown (Fig. 7E). Therefore, depletion of Akt3 does not seem to promote the differentiation of ESCs.

## DISCUSSION

Akt regulates many downstream targets that affect cell proliferation and survival. Both kinase-dependent and -independent activities of Akt have been described for cell survival (Jirmanova et al., 2002; Lianguzova et al., 2007; Vivanco et al., 2014). Delineation of the Akt regulated signaling network is difficult because there are three members in the Akt family that share structural similarities yet render isoform-specific as well as overlapping functions (Gonzalez and McGraw, 2009; Hanada et al., 2004; Manning and Cantley, 2007). We reported here for the first time a cell survival and proliferation mechanism in ESCs that is Akt3-, but not Akt1- or Akt2-, dependent. We also show that these effects of Akt3 are dependent on its kinase activity. Akt3 is predominantly activated in a number of cancers such as malignant melanoma and glioma, and plays important roles in the survival of immortalized MEFs (Liu et al., 2006; Mure et al., 2010; Stahl et al., 2004). In a recent study we reported that Akt1 and Akt3 play non-redundant roles in promoting primary MEF cell proliferation during somatic cell reprogramming (Tang et al., 2014), indicating different mechanisms governing cell growth by these two isoforms. One possible mechanism that these Akt isoforms play different functions for cell growth may be through differential cellular localization. It was reported that in several human cancer cell lines, Akt1 localizes in cytoplasm and Akt2 colocalizes with mitochondria, while Akt3 localizes in both nuclear membrane and nucleus (Santi and Lee, 2010). However, in clinical prostate cancer samples, Akt1 and -2 were reportedly localized both in cytoplasm and nucleus, whereas Akt3 was observed in cytoplasm (Le Page et al., 2006). Therefore, Akt isoform cellular localization may be cell type-specific. Exactly how different Akt isoforms localize in ESCs is certainly of great interest and worthy of further investigating.

p53 plays a central role in response to DNA damage repair resulting in cell growth arrest and apoptosis (Lakin and Jackson, 1999; Meek, 2009; Sakaguchi et al., 1998; Smith and Seo, 2002). Recent studies have also revealed a functional p53 in mouse ESCs. It was reported that in ESCs undergoing genotoxic damage, p53 protein suppresses the expression of certain pluripotency-related genes yet activates differentiation-related genes, in addition to activating the expression of Wnt-ligand genes (Lee et al., 2010; Li et al., 2012). Additionally, p53 double knockout ESCs completely, while ESCs with a single allele-mutated p53 (p53^+/R270H^ and p53^+/P275S^) partially, resisted the apoptosis induced by the genotoxic agent doxorubicin (de Vries et al., 2002). Our study for the first time established a correlation between depletion of Akt3 and the activation of p53 in ESCs at the post-transcription level.

We also demonstrated that blocking p53 expression partially rescues the apoptotic and the G1 arrest effect of Akt3 inhibition, and found that targeting p21 partially and significantly rescues the G1 arrest caused by Akt3 knockdown, in a similar fashion as p53 knockdown. Fas knockdown did not rescue apoptosis of ESCs induced by Akt3 depletion (data not shown) but showed a slight rescue to the G1 arrest, presumably due to the very low Fas expression found in ESCs (Ginis et al., 2004), and also suggests additional mechanisms parallel or downstream of p53 for the cell survival. Recently, genetic analysis on highly malignant glioma induced by Akt3 overexpression revealed a unique increase in gene expression of the DNA repair pathway (Turner et al., 2015). Our results complemented this finding and revealed that suppression of p53 pathway activity is at least partially the underlying mechanism for Akt3's role in cell survival and proliferation.

We further found that these cell propagation protective functions of Akt3 are associated with its kinase activity. It was previously shown that in primordial germ cells, enhanced Akt1 activity inhibits p53 phosphorylation at Ser20 (Kimura et al., 2008), a site necessary for p53-induced cell cycle arrest and apoptosis at the G2/M phase transition (Hirao et al., 2000; Shieh et al., 1999). Because Akt3 depletion does not impact the G2 hase in ESCs, our data indicate that Akt3 may regulate p53 activity through a mechanism other than phosphorylation of p53^Ser20^. Further study on the exact modification on p53 protein by Akt3 is of particular interest, as p53 harbors multiple phosphorylation sites for post-translational regulations (Meek and Anderson, 2009).

It is obvious that mechanisms other than p53 activation are also involved in Akt3 depletion-mediated apoptosis and cell cycle arrest. One mechanism we could potentially exclude here is the GSK3-specific inhibition by Akt3, as the 2i/LIF medium (Silva et al., 2008; Ying et al., 2008) used in our study already contains GSK3 inhibitor, and western blotting also showed an increased rather than decreased GSK3 phosphorylation in shAkt3 treated ESCs (Fig. 5C). On the other hand, our study here indicates that there is a compensatory increase of Akt1 activity to promote the survival of ESCs suffering the depletion of Akt3. We also discovered that there is a more severe effect on ESC survival by targeting both Akt1 and Akt3 than by targeting Akt3 alone, although targeting Akt1 only does not lead to cell apoptosis. Although our study here is restricted to ESCs, other cell types could well exhibit similar mechanisms and thus affect cell survival during embryo development. This correlates with a previous mouse model showing that Akt1^−/−^/Akt3^−/−^ mice died at mid-gestation stage, whereas Akt3^−/−^ mice were viable (Tschopp et al., 2005; Yang et al., 2005). A previous study also showed that a single Akt1 allele seems to be enough for the embryonic and postnatal survival of Akt2^−/−^/Akt3^−/−^ mice, albeit with series of other postnatal defects (Dummler et al., 2006). Further investigations are warranted to determine how Akt1 synergizes with Akt3 to maintain cell survivability.

Overall our results illustrated an Akt3 mediated ESC survival and G1/S-transition mechanism which involves the suppression of p53 activity. The regulation of pluripotent stem cell self-renewal is of great interest, as ESCs are promising tools for regenerative medicine. At the same time, many cancer cells exhibit ESC-specific signatures, thus making ESCs a good model for the study of the cancer cell signaling pathways (Kim and Orkin, 2011). The convergence of Akt3 and p53 pathways for ESC survival and proliferation as demonstrated here not only contributes to our understanding of pluripotent stem cell self-renewal but also has important implications in cancer research.

## MATERIALS AND METHODS

### Chemicals and expression constructs

Akt inhibitor MK2206 (MK), PI3K inhibitor LY294002, Erk inhibitor PD0329501, and GSK3β inhibitor CHIR99021 were obtained from SelleckChem (Houston, TX, USA). LIF, 100× EmbryoMax-2-mecaptoethanol, and 200× NDiff Neuro-2 medium supplement were from Millipore (Billerica, MA, USA). 50× B-27, 100× nonessential amino acids, and 100× GlutaMax supplements, 100× penicillin/streptomycin, DMEM, DMEM/F12, and neurobasal media were from Invitrogen (Grand Island, NY, USA). pLKO.1-puro, pLKO.1-scramble shRNA control, Lenti- and retro-viral packaging constructs pCMV-VSV-G, PUMVC, and psPAX2 (Stewart et al., 2003) were all obtained from Addgene (Cambridge, MA, USA). pLKO.1-shRNA constructs against mouse Akt1, Akt2, p53, Fas, and p21 were from Sigma (St. Louis, MO, USA). Constitutively active Akt3 (CA-Akt3), pLKO.1-shRNAs against Akt3 including shAkt3a and shAkt3b were described previously (Tang et al., 2014), shAkt3d were similarly constructed, with sequence information listed in Table S1. Kinase-dead Akt3 (KD-Akt3) was generated from CA-Akt3 with the lysine 177 mutated to methionine and cloned into pMCs vector from Cell Biolabs (San Diego, CA, USA). The human embryonic kidney cell line 293T for viral packaging was purchased from Invitrogen. All methods were carried out in accordance with the protocols approved by the University of Connecticut Institutional Biosafety Committee and Stem Cell Research Oversight Committee.

### Cell culture and lentiviral preparation

R1 male ESC line was obtained from ATCC. CD1 mouse embryonic fibroblasts (MEFs) were generated from E13.5 embryos as described previously (Tang et al., 2011). R1 cells were grown on mitomycin C treated CD1 MEF feeders and cultured in 2i/LIF medium with LIF (1×10^3^ units/ml) and 1× each 2-mecaptoethanol, GlutaMax, nonessential amino acids, and penicillin/streptomycin as described (Silva et al., 2009; Ying et al., 2008). For lenti- or retro-viral production, pLKO.1- or pMCs-constructs, together with packaging plasmids psPAX2 or PUMVC, and pCMV-VSV-G were co-transfected into 293T cells using Fugene 6 (Promega, Madison, WI, USA). Lenti- or retro-viruses were packaged and collected at 24 or 48 h after transfection as described previously (Tang et al., 2014, 2012).

### Apoptosis and cell cycle assays

For cell-based assays, 0.5×10^6^ R1 cells were treated in suspension with individual or a combination of different lentiviral constructs in 10% fetal bovine serum (FBS) plus LIF and plated on MEF feeders in one 12-well tissue culture plate. The next day the medium was changed to 2i/LIF and cells were allowed to grow for 48 h before analysis. For apoptosis assay, cells were trypsinized and treated with an Annexin V-FITC apoptosis detection kit (Sigma). Briefly, after trypsinization cells from each condition were resuspended in 0.5 ml 1× binding buffer and incubated with 0.125 μg/ml Annexin V-FITC (Annex) and 0.1 μg/ml propidium iodide (PI) for 30 min. Cells were then analyzed with a BD FACSCalibur flow cytometer with fluorescence excitation at 488 nm, or a BD LSRFortessa X-20 with excitation of 488 nm and 561 nm. Annexin V single positive and Annexin/PI double-positive cell gates were established based on populations seen in singly labeled samples. For cell cycle assay, cells were resuspended in 0.5 ml 2% FBS in PBS, then treated with a final concentration of 0.2% Triton X-100, 50 µg/ml PI (Sigma), and 200 µg/ml DNase-free RNase (Thermo Fisher Scientific) for 20 min in the dark, before flow cytometry. The cell cycle was analyzed using Watson algorithm associated with the FlowJo software (http://www.flowjo.com/).

### Immunostaining

R1 cells were grown on 12 mm glass coverslips (Thermo Fisher Scientific) in 6-well plates seeded with CD1 MEFs as feeders, and treated by lentiviruses as described in the Apoptosis and cell cycle assays section. Cells were fixed in 4% paraformaldehyde with 1% sucrose in PBS for 15 min at room temperature. The cell membranes were permeabilized with 0.5% Triton X-100 in PBS-T, then incubated for 2 h at 37°C in 5% donkey or goat serum with mouse anti-SSEA1 IgM (1:100, Abcam, Cambridge, MA, USA) or rabbit anti-p53 IgG (1:100, Cell Signaling, Danvers, MA, USA), washed in PBS-T, and then incubated with Alexa Fluor 594 conjugated donkey anti-rabbit or goat anti-mouse secondary antibodies (1:500, Invitrogen). After the washing, cells were counterstained with DAPI and mounted under coverslips. Fluorescence images were taken using a Nikon A1R Spectral confocal microscope and signal densities were analyzed using the ImageJ software (Schneider et al., 2012).

### Western blotting

R1 cells were grown in 6-well plates seeded with CD1 MEFs as feeders, and treated by lentiviruses as described in the Apoptosis and cell cycle assays section. Total cellular proteins were then extracted using RIPA buffer (Thermo Fisher Scientific) with 1× proteinase and phosphatase inhibitors (Thermo Fisher Scientific). Proteins were quantified with a BCA-Quantification kit (Thermo Fisher Scientific), and subjected to 10% SDS-PAGE gel electrophoresis using BioRad mini-gel system and subsequently transferred to PVDF membranes.

The blotted membranes were then blocked with 5% non-fat dry milk in TBS-T and incubated with primary antibodies at 4°C overnight. The antibodies used were as follows: anti-GAPDH (1/2500, Abcam). Antibodies against p53, Mdm2, pMdm2, and pGSK3β (1/1000 for all) were from Cell Signaling. All Akt isoforms, phospho-Akt Ser473, and pan-Akt were detected using Akt isoform antibody sampler kit from Cell Signaling. Membranes were then washed and blotted with HRP conjugated goat anti-mouse or goat anti-rabbit secondary antibodies (1:5000, Santa Cruz Biotechnology, Santa Cruz, CA, USA). Chemiluminescence was detected either using Pierce ECL Western-Blot Substrate (Thermo Fisher Scientific) and X-ray film exposure, and quantified using the ImageJ software (Schneider et al., 2012), or detected and quantified using BioRad Gel Doc XR System (Hercules, CA, USA).

### Quantitative real time-reverse transcription polymerase chain reaction (qRT-PCR)

Total RNA was extracted using the RNeasy Extraction kit (Qiagen), and reverse transcribed using an iScript Advanced cDNA Synthesis Kit (Bio-Rad). qRT-PCR was performed using iTaq Universal SYBR Green Supermix (Bio-Rad) and the ABI 7500 Fast instrument. Sequence information for the specific primers for qPCR were listed in Table S2. The data were analyzed using the 7500 software version 2.0.2 provided with the instrument. All values were normalized with GAPDH as the internal control and relative mRNA expressions were quantified against control lentivirus transduced R1 cells as the reference.

### Statistical analysis

Data were analyzed using one way ANOVA with Tukey's multiple comparisons, or the Student's *t*-test. All experiments were repeated at least two times (*N*≥2). Figures were presented as mean±s.d. *P<0.05 or **P<0.01 was considered significantly different.
